# Hospitalization for COVID-19 is associated with a higher risk of subsequent hospitalization for psychiatric disorders: A French nationwide longitudinal study comparing hospitalizations for COVID-19 and for other reasons

**DOI:** 10.1192/j.eurpsy.2022.2331

**Published:** 2022-10-21

**Authors:** Valentina Decio, Philippe Pirard, Baptiste Pignon, Olivier Bouaziz, Vittorio Perduca, Francis Chin, Yann Le Strat, Jonathan Messika, Viviane Kovess-Masfety, Emmanuelle Corruble, Nolwenn Regnault, Sarah Tebeka

**Affiliations:** 1 Non-Communicable Diseases and Trauma Division, Santé publique France, the National Public Health Agency, F-94415 Saint-Maurice, France; 2 Université Paris-Est-Créteil (UPEC), AP-HP, Hôpitaux Universitaires “H. Mondor”, DMU IMPACT, INSERM, IMRB, Translational Neuropsychiatry, F-94010 Créteil, France; 3 Fondation FondaMental, Créteil, France; 4 Université Paris Cité, CNRS, MAP5, F-75006 Paris, France; 5 Data Science Division, Santé publique France, the National Public Health Agency, F-94415 Saint-Maurice, France; 6 APHP.Nord—Université Paris Cité, Hôpital Bichat-Claude Bernard, Service de Pneumologie B et Transplantation Pulmonaire, Paris, France; 7 Physiopathology and Epidemiology of Respiratory Diseases, UMR1152 INSERM and Université de Paris, Paris, France; 8 LPPS, Université de Paris, Paris, France; 9 CESP, MOODS Team, INSERM UMR 1018, Faculté de Médecine, Université Paris-Saclay, Le Kremlin Bicêtre F-94275, France; 10 Service Hospitalo-Universitaire de Psychiatrie de Bicêtre, Hôpitaux Universitaires Paris-Saclay, Assistance Publique-Hôpitaux de Paris, Hôpital de Bicêtre, Le Kremlin Bicêtre F-94275, France

**Keywords:** COVID-19, epidemiology, hospitalization, psychiatric disorders, SNDS

## Abstract

**Introduction:**

Although COVID-19 has been associated with psychiatric symptoms in patients, no study to date has examined the risk of hospitalization for psychiatric disorders after hospitalization for this disease.

**Objective:**

We aimed to compare the proportions of hospitalizations for psychiatric disorders in the 12 months following either hospitalization for COVID-19 or hospitalization for another reason in the adult general population in France during the first wave of the current pandemic.

**Methods:**

We conducted a retrospective longitudinal nationwide study based on the national French administrative healthcare database.

**Results:**

Among the 2,894,088 adults hospitalized, 96,313 (3.32%) were admitted for COVID-19. The proportion of patients subsequently hospitalized for a psychiatric disorder was higher for COVID-19 patients (11.09 vs. 9.24%, OR = 1.20 95%CI 1.18–1.23). Multivariable analyses provided similar results for a psychiatric disorder of any type and for psychotic and anxiety disorders (respectively, aOR = 1.06 95%CI 1.04–1.09, aOR = 1.09 95%CI 1.02–1.17, and aOR = 1.11 95%CI 1.08–1.14). Initial hospitalization for COVID-19 in intensive care units and psychiatric history were associated with a greater risk of subsequent hospitalization for any psychiatric disorder than initial hospitalization for another reason.

**Discussion:**

Compared with hospitalizations for other reasons, hospitalizations for COVID-19 during the first wave of the pandemic in France were associated with a higher risk of hospitalization for a psychiatric disorder during the 12 months following initial discharge. This finding should encourage clinicians to increase the monitoring and assessment of psychiatric symptoms after hospital discharge for COVID-19, and to propose post-hospital care, especially for those treated in intensive care.

## Introduction

From the beginning of the COVID-19 pandemic, concerns have been raised about its impact on the mental health of general populations [1, 2]. Early in the pandemic, data for general populations worldwide confirmed a significant increase in depressive and anxiety symptoms between the pre- and mid-pandemic periods [3].

This impact seemed to be stronger in individuals infected by the disease than in those not infected [4, 5]. More specifically, several studies have indicated that COVID-19 patients have more symptoms of anxiety, post-traumatic stress, depression, insomnia and suicidal thoughts [4, 6–11]. One hypothesis for this is that direct viral infection of the central nervous system or through immune response may cause psychiatric disorders [8, 12, 13].

Severe COVID-19 infection, which results in intensive care, can have an strong impact on mental health [14]. Data on the emergence of psychiatric symptoms after hospitalization for COVID-19 highlight the prevalence of several disorders including posttraumatic stress symptoms, anxiety, and depression, which can continue up to 6 months after discharge [6, 15–17]. This is especially true for persons with severe COVID-19 infection who have acute respiratory distress syndrome and require therapy in an intensive care unit (ICU) [18, 19]. A systematic review performed several years before the COVID-19 pandemic found that nearly 30% of ICU patients had significant depressive symptoms during the first year after hospital discharge [20]. A recent meta-analysis confirmed this finding, showing that a third of ICU patients experienced persistent anxiety symptoms during their first year of recovery [21].

However, it remains unknown whether hospitalizations for COVID-19, compared with hospitalizations for a different reason, are associated with a higher risk of subsequent severe psychiatric disorders and related hospitalizations. The present study aimed to compare the proportion of hospitalizations for psychiatric disorders in the 12 months following discharge from hospital for either COVID-19 or for another reason in the general adult population in France during the first wave of the SARS-CoV-2 pandemic.

## Methods

### Data sources

The present study used data from France’s national administrative healthcare database *Système National des Données de Santé* (SNDS), which covers almost the country’s entire population of 67 million inhabitants (i.e., 66.3 million in 2020). Although widely used in France to conduct various types of research, it has not been exploited to any great degree in the field of psychiatric research despite the great potential it offers [22].

One of the components of the SNDS is the *Programme de médicalisation des systèmes d’information* (PMSI), which is a database providing admission and discharge information for all public and private hospital stays throughout the country. The PMSI itself comprises four separate databases, three of which were used for the present study as follows: (a) the *PMSI en Médecine, chirurgie, obstétrique et odontologie* (PMSI-MCO) which exhaustively records all medical, surgical, and obstetric-based hospitalizations (including ICU settings); (b) the *Recueil d’information medicalisée en psychiatrie* (RIM-P), which collects exhaustive data on hospitalizations in psychiatric wards, and (c) the *PMSI en soins de suite et de réadaptation* (PMSI-SSR), which exhaustively records all data on hospitalizations for follow-up and rehabilitation care. For all these hospitalization types, medical diagnoses are coded according to the International Classification of Diseases 10th edition (ICD-10), whereas the main medical and surgical procedures performed are coded according to the *Classification Commune des Actes Medicaux* (CCAM, or Common Classification of Medical Procedures).

Different hospitalizations for the same patient can be identified thanks to a unique patient identification number [23].

For the present study, we also used the *Cartographie des Pathologies et des Dépenses* (Diseases and Expense Mapping) database to identify the presence of a psychiatric history over the 5 years preceding the study period for each individual included. Its medical algorithms are based on data from the SNDS and are publicly available in French [24].

### Study design and participants

This retrospective longitudinal study aimed to compare the risk of hospitalization for psychiatric disorders during the 12 months following hospital discharge from a medical, surgical, or obstetrics ward in adult patients (i.e., 18 years or over) who had either been hospitalized for COVID-19 or for another reason between January 1, 2020 and June 30, 2020 in metropolitan France.

For each individual, a reference hospital stay was selected. For patients with more than one hospitalization during the study period, if at least one of these was COVID-19 related (*N* = 85,514), then it was considered the reference hospital stay. For patients with two or more COVID-19-related hospitalizations (*N* = 7,521), the reference stay was the one where the most intensive level of clinical care was provided. This care intensity criterion was also adopted to decide on the reference stay for patients with two or more hospitalizations for a pathology other than COVID-19.

We followed the most recent coding guidelines from the *Agence technique de l’information sur l’hospitalization* (ATIH, Technical Agency for Information on Hospitalization) (ATIH, Consignes de codage COVID-19, 2021) to identify COVID-19-related hospitalizations [64]. The ATIH is a public establishment which provides data on the different areas of hospital activity we investigated here (i.e., medical, surgical, obstetrics procedures, etc.). More specifically, all patients with an ICD-10 diagnosis code *U07.1*, *U07.10*, *U07.11*, *U07.12*, *U07.14*, *U07.15* whether as a primary, related, or associated diagnosis, were considered to have been hospitalized for COVID-19.

### Outcomes

The main outcome was hospitalization for a psychiatric disorder of any type in the 12-month period following discharge for the reference hospital stay. We looked for inpatient and outpatient admissions in medical, surgical, and obstetrics wards (through the PMSI-MCO database), in psychiatric wards (RIM-P database), as well as in follow-up care and rehabilitation facilities (PMSI-SSR database) that presented the following ICD-10 codes as a primary or related diagnosis:

1. Schizophrenia, schizotypal and delusional disorders (*F20–F29*) *(Psychotic disorders*), including schizophrenia spectrum and other psychotic disorders.
2. Mood [affective] disorders (*F30–F39*) (*Mood disorders*), including depression and bipolar disorders.
3. Neurotic, stress-related and somatoform disorders (*F40–F49*) (*Anxiety disorders*), including anxiety, obsessive–compulsive disorder (OCD), PTSD, and somatoform disorders.
4. Behavioral syndromes associated with physiological disturbances and physical factors (*F50–F59*) (*Behavioral syndromes*), including eating disorders, nonorganic sleep disorders, and sexual dysfunctions.
5. Disorders of adult personality and behavior (*F60–F69*) (*Personality disorders*).

The secondary outcomes were hospital admissions for each specific psychiatric disorder.

### Variables of interest

#### Sociodemographic characteristics

Age, sex, and region of residence were the demographic variables considered. Age was calculated from the individual’s year of birth and categorized according to one of four age groups (18–39 years, 40–59 years, 60–74 years, and 75+ years). The French Deprivation Index (Fdep), developed by the *Centre d’épidémiologie sur les causes médicales de décès* (CépiDc) was used as a measure of socioeconomic status. This indicator is based on the median household income, the percentage of higher education graduates in the population over 15 years old, the percentage of manual workers in the labor force, and the unemployment rate of the individual’s city of residence [25]. It has already been used in other studies focusing on COVID-19 [26].

#### Psychiatric history

The *Cartographie des Pathologies et des Dépenses* database (8th version) was used to assess whether each individual had a psychiatric history in the 5 years preceding the study period; this was summarized through a dichotomous variable (yes/no). An individual was considered to have had a psychiatric history for a specific year if one of the following was found in the SNDS for that year: (a) declaration by a healthcare professional that the patient had a psychiatric disorder officially recognized as a long-term disease; (b) hospitalization(s) for psychiatric pathologies in a psychiatric (RIM-P) and/or nonpsychiatric hospital or healthcare center (MCO or SSR) during at least one of the previous 2 years; (c) hospitalization(s) for these same reasons in a psychiatric (RIM-P) and/or nonpsychiatric health establishment (MCO or SSR) during at least one of the last 5 years (*n* to *n* − 4) and receiving prescribed psychotropic drugs on at least three different occasions during year *n* [24].

#### Characteristics of the reference hospital stay

Median duration (in days) and level of clinical care were the elements used to characterize the reference hospital stay. We defined three levels of care intensity for hospitalizations for COVID-19 and for other reasons. These three levels were defined according to care provided in general for different degrees of COVID-19 severity (see the list of procedures used to define the levels of clinical care in Supplementary Table S1):

1. The first level corresponded to patients with the mildest level of respiratory difficulty and admitted to a general hospital ward; they required no or low-flow oxygen (up to 15 L/min).
2. The second level corresponded to patients admitted to an ICU, irrespective of the level (i.e., type and flowrate) of oxygen supply therapy, and patients who received high-flow nasal oxygen or noninvasive ventilation.
3. The maximum level of care corresponded to patients who were hospitalized in an ICU and required at least invasive ventilatory support.

### Statistical analysis

Table 1 describes the characteristics of our study population, and the reasons for their initial hospitalization (i.e., COVID-19 or other reason). We used logistic regression models to estimate and compare the risks of hospitalization for psychiatric disorders in individuals initially admitted for COVID-19 and in those initially admitted for another reason.

**Table 1.** Comparison of patients hospitalized for COVID-19 and those hospitalized for another reason.

	Total(*N* = 2,894,088)	Hospitalizations for COVID-19(*N* = 96,313)	Hospitalizations for another reason (*N* = 2,797,775)	*p-*value
Sex				<0.0001
Male	1,270,314 (43.89)	50,699 (52.64)	1,219,615 (43.59)	
Female	1,623,774 (56.11)	46,614 (47.36)	1,578,160 (56.41)	
Age (years)				<0.0001
18–39	669,227 (23.12)	7,940 (8.24)	661,287 (23.64)	
40–59	612,000 (21.15)	20,701 (21.49)	591,299 (21.13)	
v60–74	750,466 (25.93)	26,436 (27.45)	724,030 (25.88)	
75+	862,395 (29.8)	41,236 (42.81)	821,159 (29.35)	
Region				<0.0001
Île-de-France	435,139 (15.04)	33,999 (35.30)	401,140 (14.34)	
Centre-Val de Loire	115,194 (3.98)	2,936 (3.05)	112,258 (4.01)	
Bourgogne-Franche-Comté	138,196 (4.78)	4,872 (5.06)	133,324 (4.77)	
Normandy	157,841 (5.45)	2,736 (2.84)	155,105 (5.54)	
Hauts-de-France	287,055 (9.92)	9,754 (10.13)	277,301 (9.91)	
Grand Est	251,670 (8.70)	14,752 (15.32)	236,918 (8.47)	
Pays de la Loire	165,845 (5.73)	2,890 (3)	162,955 (5.82)	
Brittany	156,072 (5.39)	1,487 (1.54)	154,585 (5.53)	
Nouvelle-Aquitaine	295,320 (10.20)	3,169 (3.29)	292,151 (10.44)	
Occitanie	273,117 (9.44)	3,699 (3.84)	269,418 (9.63)	
Auvergne-Rhône-Alpes	357,598 (12.36)	10,237 (10.63)	347,361 (12.42)	
Provence-Alpes-Côte d’Azur	245,258 (8.47)	5,523 (5.73)	239,735 (8.57)	
Corsica	15,783 (0.55)	259 (0.27)	15,524 (0.55)	
Social deprivation index (quintiles)				<0.0001
1 (least deprived)	476,939 (16.74)	22,591 (23.76)	454,348 (16.5)	
2	541,042 (18.99)	16,554 (17.41)	524,488 (19.05)	
3	593,564 (20.84)	16,315 (17.16)	577,249 (20.96)	
4	613,369 (21.53)	17,119 (18)	596,250 (21.65)	
5 (most deprived)	623,615 (21.89)	22,507 (23.67)	601,108 (21.83)	
Unknown	45,559 (1.57)	1,227 (1.27)	44,332 (1.49)	
Psychiatric history				<0.0001
No	2,574,477 (88.96)	83,224 (86.41)	2,491,253 (89.04)	
Yes	319,611 (11.04)	13,089 (13.59)	306,522 (10.96)	
Duration of reference hospitalization (days)—median (IQ)	3 (1–6)	7 (3–14)	3 (1–6)	<0.0001
Level of clinical care				<0.0001
General hospital ward (medical, surgery, obstetrics)	2,528,207 (87.36)	72,839 (75.63)	2,455,368 (87.76)	
Intensive care unit	301,188 (10.41)	13,557 (14.08)	287,631 (10.28)	
Intensive care unit withinvasive procedures	64,693 (2.24)	9,917 (10.30)	54,776 (1.96)	

Six nested models were successively performed for each outcome:

1. Model 1 described the crude associations between outcomes, and the reason for initial hospitalization (COVID-19 versus another reason);
2. Model 2: Model 1 plus socio-demographic variables;
3. Model 3: Model 2 plus psychiatric history;
4. Model 4: Model 3 plus the characteristics of the reference hospital stay (median duration and level of clinical care).

We searched for multiplicative interactions between psychiatric history and initial hospitalization for COVID-19 or for any other reason. We also searched for multiplicative interactions between the level of clinical care received and type of initial hospitalization. We also looked for multiplicative interactions between age groups and the type of initial hospitalization. For each statistically significant interaction term, we subsequently conducted stratified analyses as follows:

1. Model 5: Model 4 with no adjustment for psychiatric history, but stratified by this dichotomous (yes/no) variable.
2. Model 6: Model 4 with no adjustment for the intensity of clinical care, but stratified according to the three different levels of this variable.
3. Model 7: Model 4 with no adjustment for the age groups, but stratified according to the four different levels of this variable.

For all the different multivariable models, we conducted a power analysis for the effect of COVID-19 hospitalization on the different outcomes, for a type 1 error of 5% and a grid range of odds ratios. These analyses were based on a formula (10) for the Wald test in logistic regression [27].

Statistical analyses were performed using SAS software, Version 7.1 (SAS Institute Inc., Cary, NC, USA.).

### Role of funding source, regulatory approval and ethical aspects

This research was conducted as part of the surveillance activities of the French agency for public health (Santé publique France [SpF]). It did not receive any specific grant from funding agencies in the public, commercial, or not-for-profit sectors.

The SNDS comprises a set of strictly pseudonymized databases. By law, SpF has permanent regulatory access to SNDS data for the performance of its missions (article L.1461-3 and R1461-13 and following of the French public health code [28]). Access to personal data in these systems for research purposes is subject to obtaining an authorization from the *Commission nationale de l’informatique et des libertés* (CNIL, National Commission on Information Technology and Civil Liberties), after advice from the *Comité éthique et scientifique pour les recherches, les études et les évaluations dans le domaine de la santé* (CESREES, Ethics and Scientific Committee for Research, Studies and Evaluation in the field of health).

## Results

### Cohort description

**Figure 1.**
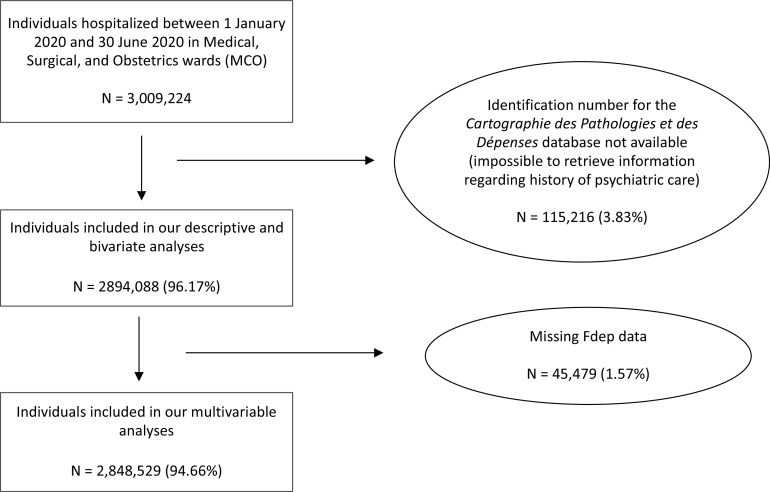
Flow-chart.

A total of 3,009,224 individuals were admitted as hospital inpatients in medical (including in ICU), surgical, and obstetrics wards at least once between January 1, 2020 and June 30, 2020 in metropolitan France (Figure 1). Among these, reference hospital stays were identified for 2,894,088, of which 96,313 (3.33%) were hospitalizations for COVID-19 and 2,979,775 (96.67%) for a different reason (see Supplementary Figure S1 for details). The cohort’s characteristics are presented in Table 1. Although women represented the majority of the sample (56.11%), men were more numerous among COVID-19 patients than among patients hospitalized for another reason (52.64 vs. 43.59%). COVID-19 patients were older (42.81 vs. 29.35% in the 75+ age group, 27.45 vs. 25.88% in the 60- to 74-years old group) and their median length of stay was twice as long as that of other patients (7 days, interquartile range 3 to 14 days vs. 3 days, interquartile range 1 to 6 days, respectively). The proportion of individuals with a psychiatric history was higher in COVID-19 patients (13.59 vs. 10.96%), in patients admitted to ICU, in those who received noninvasive ventilation (14.08 vs. 10.28%), and in ICU patients who received invasive ventilation (10.3 vs. 1.96%).

**Figure 2.**
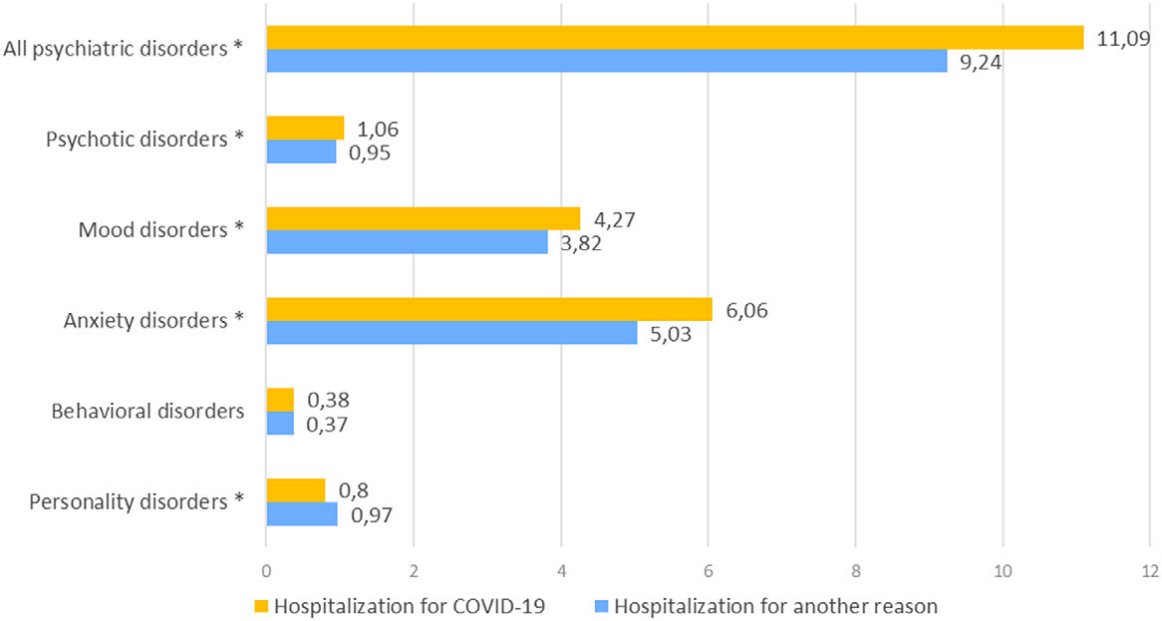
Comparison of reasons of psychiatric hospitalization in patients previously hospitalized for COVID-19 *vs.* for another medical reason (percentages of total hospitalizations for each category of psychiatric disorder). *p-value of the chi-square test <0.05.

Over the 12-month period following their reference hospital stay, 269,251 (9.3%) individuals were re-hospitalized at least once for a psychiatric disorder. These comprised 11.09% of the patients initially hospitalized for COVID-19, and 9.24% of those initially hospitalized for another medical reason. Of the 9.3% re-hospitalized, 5.07% (*n* = 146,653) were admitted for anxiety disorders and 3.83% (*n* = 110,976) for mood disorders. These figures translated into 6.06 and 4.27%, respectively, of the 11.09% initially hospitalized for COVID-19, and 5.03 and 3.82%, respectively, of the 9.24% hospitalized for another reason. In other words, COVID-19 patient percentages were higher for both types of disorder. The same was found for psychotic disorders (1.06 vs. 0.95%). Conversely, the proportion of hospitalizations for personality disorders was lower among COVID-19 patients (0.8 vs. 0.97%). No difference was observed for behavioral syndromes between the two groups (Figure 2 and Supplementary Table S2).

### Risk of hospitalization for psychiatric disorders

#### Hospitalization for a psychiatric disorder of any type

Associations between the risk of admission for a psychiatric disorder of any type during the 12 months following the reference hospitalization and sociodemographic factors, psychiatric history, characteristics of the reference hospitalization (i.e., median length of stay and level of clinical care) are shown in Table 2.

**Table 2.** Odds-ratio (OR), adjusted odds-ratio (aOR) and 95% confidence interval (95%CI) for the risk of subsequent hospitalization for a psychiatric disorder of any type over the 12-month period after initial hospital discharge, for patients hospitalized for COVID-19 versus those hospitalization for another reason, in all adult patients hospitalized in metropolitan France the first half of 2020.

	Model 1^a^		Model 2^b^		Model 3^c^		Model 4^d^	
	OR (95%CI)	*p*-value	aOR (95%CI)	*p*-value	aOR (95%CI)	*p*-value	aOR (95%CI)	*p*-value
Hospitalization for COVID-19								
No	1		1		1		1	
Yes	1.20 [1.18–1.23]	<0.0001	1.14 [1.12–1.17]	<0.0001	1.06 [1.04–1.09]	<0.0001	0.93 [0.91–0.95]	<0.0001
Sex			1		1		1	
Male			1.25 [1.24–1.26]	<0.0001	1.10 [1.09–1.11]	<0.0001	1.11 [1.1–1.12]	<0.0001
Female								
Age (years)								
18–39			1		1		1	
40–59			2.04 [2.01–2.07]	<0.0001	1.59 [1.57–1.61]	<0.0001	1.55 [1.53–1.58]	<0.0001
60–74			1.75 [1.73–1.78]	<0.0001	1.48 [1.46–1.5]	<0.0001	1.39 [1.37–1.41]	<0.0001
75+			2.39 [2.36–2.42]	<0.0001	1.97 [1.95–2]	<0.0001	1.81 [1.78–1.83]	<0.0001
Social deprivation index (quintiles)								
1 (least deprived)			1		1		1	
2			0.94 [0.93–0.96]	<0.0001	0.95 [0.94–0.96]	<0.0001	0.95 [0.93–0.96]	<0.0001
3			1 [0.98–1.01]	0.4631	0.97 [0.96–0.99]	<0.0001	0.96 [0.95–0.98]	<0.0001
4			1 [0.98–1.01]	0.8437	0.97 [0.97–0.99]	0.0002	0.96 [0.95–0.98]	<0.0001
5 (most deprived)			1.01 [1–1.03]	0.1188	0.98 [0.97–0.99]	0.0113	0.97 [0.95–0.98]	<0.0001
Psychiatric history								
No					1		1	
Yes					7.65 [7.58–7.72]	<0.0001	7.64 [7.57–7.71]	<0.0001
Duration of reference hospitalization (days)—mean(SD)							1.02 [1.02–1.02]	<0.0001
Level of clinical care								
General hospital ward (medical, surgery, obstetrics)							1	
Intensive care unit							1.05 [1.03–1.06]	<0.0001
Intensive care unit with invasive procedures							1.16 [1.13–1.2]	<0.0001

a No adjustment.

b *Odds-ratio* adjusted for socio-demographic characteristics: sex, age, region and social deprivation index.

c *Odds-ratio* adjusted for socio-demographic characteristics and psychiatric history.

d *Odds-ratio* adjusted for socio-demographic characteristics, psychiatric history and characteristics of the reference hospitalization: duration of hospitalization (days) and level of clinical care.

A significant positive association was found between hospitalization for COVID-19 during the first semester of 2020 and subsequent hospitalization for a psychiatric disorder of any type (Model 1: OR = 1.20, 95%CI 1.18–1.23). This association remained positive after adjusting for sociodemographic factors (Model 2: adjusted OR [aOR] = 1.14, 95%CI 1.12–1.17), but was attenuated after adjustment for psychiatric history (Model 3: aOR = 1.06, 95%CI 1.04–1.09). A slightly negative association was found after adjusting for the characteristics of the reference hospitalization (Model 4: aOR = 0.93, 95%CI 0.91–0.95). In the latter model, psychiatric history was by far the factor most associated with the risk of hospitalization for a psychiatric disorder (Model 3: aOR = 7.65 95%CI 7.58–7.72). Patient age and level of clinical care were also associated, particularly in the 75+ age group (Model 4: aOR = 1.81, 95%CI 1.78–1.83), in the ICU or noninvasive ventilatory support group (Model 4: aOR = 1.05, 95%CI 1.04–1.07), and in the ICU with invasive ventilatory support group (Model 4: aOR = 1.16, 95%CI 1.13–1.2).

#### Secondary outcomes: results according to specific psychiatric disorder

A similar slightly negative association was also, that is, in addition to disorders globally observed for mood disorders (Model 4: aOR = 0.87, 95%CI 0.84–0.9) and for anxiety disorders (Model 4: aOR = 0.98, 95%CI 0.95–1) (Table 3). Psychiatric history, age (75+ age group), level of clinical care (ICU with invasive ventilation group) and female sex were the factors most associated with the risk of hospitalization for mood and anxiety disorders following an hospitalization for COVID-19 (Supplementary Tables S4 and S5).

**Table 3.** Odds ratio (OR), adjusted odds ratio (aOR) and 95% confidence interval (95%CI) for the risk of subsequent hospitalization for psychotic disorders, mood disorders, anxiety disorders, and personality disorders over the 12-month period after initial hospital discharge, for patients hospitalized for COVID-19 versus those hospitalization for another reason, in all adult patients hospitalized in metropolitan France the first half of 2020.

	Model 1^a^		Model 2^b^		Model 3^c^		Model 4^d^	
	OR (95%CI)	*p*-value	aOR (95%CI)	*p*-value	aOR (95%CI)	*p*-value	aOR (95%CI)	*p*-value
Psychotic disorders	1.11 [1.05–1.19]	<0.0001	1.16 [1.09–1.24]	<0.0001	1.09 [1.02–1.17]	0.009	1.06 [0.99–1.14]	0.09
Mood disorders	1.12 [1.09–1.16]	<0.0001	1.07 [1.03–1.1]	0.0001	0.97 [0.94–1]	0.078	0.87 [0.84–0.9]	<0.0001
Anxiety disorders	1.22 [1.18–1.25]	<0.0001	1.18 [1.14–1.21]	<0.0001	1.11 [1.08–1.14]	<0.0001	0.98 [0.95–1.01]	0.154
Personality disorders	0.82 [0.76–0.88]	<0.0001	0.92 [0.86–0.99]	0.032	0.86 [0.8–0.92]	0.0001	0.82 [0.76–0.88]	<0.0001

a No adjustment.

b *Odds-ratio* adjusted for socio-demographic characteristics: sex, age, region and social deprivation index.

c *Odds-ratio* adjusted for socio-demographic characteristics and psychiatric history.

d *Odds-ratio* adjusted for socio-demographic characteristics, psychiatric history and characteristics of the reference hospitalization: duration of hospitalization (days) and level of clinical care.

With regard to the risk of subsequent hospitalization for psychotic disorders, the stronger association with persons initially hospitalized for COVID-19 remained positive but became slightly nonsignificant after adjusting for all the co-variables (Model 4: aOR = 1.06, 95%CI 0.99–1.14) (Table 3). Psychiatric history and level of clinical care remained the two factors most associated with this risk of hospitalization for psychotic disorders (Supplementary Table S3).

Conversely, a negative association was found between hospitalization for COVID-19 and the risk of subsequent hospitalization for a personality disorder (Model 4: aOR = 0.82; 95%CI 0.76–0.88) in all the models (Table 3). Psychiatric history and level of clinical care (ICU with invasive ventilation group) were the two factors most associated with the risk of hospitalization for personality disorders (Supplementary Table S6).

### Interactions and stratified analyses

We found significant multiplicative interactions between hospitalization for COVID-19 or for another reason and level of clinical care received. After stratification by level of clinical care, multivariable analyses showed a similar association for the risk of subsequent hospitalization for a psychiatric disorder of any type (primary outcome) in the ICU with invasive ventilatory support group (Model 6: aOR = 1.16, 95%CI 1.08–1.25) (Table 4), and for anxiety disorders (secondary outcome) (aOR = 1.36, 95%CI 1.24–1.48) (Supplementary Table S8).

**Table 4.** Adjusted odds ratio (aOR) and 95% confidence interval (95%CI) for the risk of subsequent hospitalization a psychiatric disorder of any type over the 12-month period after initial hospital discharge, for patients hospitalized for COVID-19 versus those hospitalization for another reason, in all adult patients hospitalized in metropolitan France the first half of 2020: Model 5, stratified by psychiatric history versus no psychiatric history using fully-adjusted analysis (Model 4), and Model 6, stratified by level of clinical care using model adjusted for all variables (Model 4).

	Psychiatric history		No psychiatric history		General hospital ward		Intensive care unit		Intensive care unit with invasive procedures	
	aOR (95%CI)	*p*-value	aOR (95%CI)	*p*-value	aOR (95%CI)	*p*-value	aOR (95%CI)	*p*-value	aOR (95%CI)	*p*-value
Hospitalization for COVID-19										
No	1		1		1		1		1	
Yes	0.85 [0.82–0.89]	<0.0001	1.03 [1–1.06]	0.0474	0.91 [0.89–0.93]	<0.0001	0.88 [0.82–0.94]	<0.0001	1.16 [1.08–1.25]	<0.0001
Sex										
Male	1		1		1		1		1	
Female	1.04 [1.03–1.06]	<0.0001	1.26 [1.24–1.27]	<0.0001	1.07 [1.06–1.08]	<0.0001	1.44 [1.4–1.48]	<0.0001	1.59 [1.51–1.67]	<0.0001
Age (years)										
18–39	1		1		1		1		1	
40–59	0.91 [0.89–0.93]	<0.0001	1.96 [1.92–2]	<0.0001	1.6 [1.58–1.63]	<0.0001	0.92 [0.88–0.97]	0.0016	0.74 [0.68–0.81]	<0.0001
60–74	0.71 [0.69–0.73]	<0.0001	2.01 [1.97–2.05]	<0.0001	1.46 [1.44–1.48]	<0.0001	0.77 [0.73–0.81]	<0.0001	0.5 [0.46–0.55]	<0.0001
75+	0.53 [0.51–0.54]	<0.0001	3.29 [3.23–3.35]	<0.0001	1.94 [1.91–1.96]	<0.0001	0.85 [0.81–0.9]	<0.0001	0.4 [0.36–0.43]	<0.0001
Social deprivation index (quintiles)										
1 (least deprived)	1		1		1		1		1	
2	0.94 [0.91–0.96]	<0.0001	0.95 [0.93–0.97]	<0.0001	0.95 [0.93–0.96]	<0.0001	0.94 [0.9–0.98]	0.0063	0.94 [0.86–1.03]	0.0502
3	0.94 [0.92–.097]	<0.0001	0.96 [0.95–0.98]	<0.0001	0.96 [0.95–0.98]	<0.0001	0.97 [0.92–1.01]	0.1169	0.9 [0.82–0.98]	0.017
4	0.9 [0.88–0.92]	<0.0001	0.98 [0.97–1]	0.0709	0.97 [0.95–0.98]	<0.0001	0.94 [0.9–0.98]	0.0047	0.88 [0.81–0.96]	0.0004
5 (most deprived)	0.89 [0.87–0.92]	<0.0001	0.99 [0.97–1.01]	0.4146	0.97 [0.96–0.99]	0.001	0.94 [0.9–0.98]	0.0041	0.89 [0.81–0.96]	0.0008
History of psychiatric care										
No					1		1		1	
Yes					7.69 [7.62–7.77]	<0.0001	6.78 [6.59–6.98]	<0.0001	6.48 [6.12–6.85]	<0.0001
Duration of reference hospitalization (days)—mean (SD)	1.01 [1.01–1.01]	<0.0001	1.03 [1.03–1.03]	<0.0001	1.02 [1.02–1.03]	<0.0001	1.02 [1.02–1.02]	<0.0001	1.01 [1.01–1.01]	<0.0001
Level of clinical care										
General hospital ward (medical, surgery, obstetrics)	1		1							
Intensive care unit	1.05 [1.03–1.08]	<0.0001	1.01 [0.99–1.02]	0.5234						
Intensive care unit with invasive procedures	1.2 [1.14–1.26]	<0.0001	1.06 [1.03–1.1]	0.0004						

With regard to the risk of hospitalization for a psychiatric disorder of any type, we also found significant multiplicative interactions between both hospitalization for COVID-19 or for another reason and psychiatric history. Stratified multivariable analyses showed a negative association between initial hospitalization for COVID-19 and the risk of subsequent hospitalization for a psychiatric disorder of any type in patients with a psychiatric history (Model 5: aOR = 0.85, 95%CI 0.82–0.89), and a positive association in those with no psychiatric history (Model 5: aOR = 1.03, 95%CI 1–1.06) (Table 4). Similar results were found for the risk of hospitalization for anxiety disorders (Supplementary Table S8).

We found significant multiplicative interactions between hospitalization for COVID-19 or for another reason and age groups. After stratification by age groups, multivariable analyses showed a significant positive association between initial hospitalization for COVID-19 and the risk of subsequent hospitalization for a psychiatric disorder of any type in the 60–74 age group (Model 7: aOR = 1.10, 95%CI 1.03–1.74), whereas the association was significantly negative for the youngest (18–39) and oldest (75+) age groups (Model 7: aOR = 0.82, 95%CI 0.70–0.96 and aOR = 0.94, 95%CI 0.90–0.97, res pe ctively) (Table 5 and Supplementary Table S10). Moreover, different results were found for different types of disorders. In particular, subsequent hospitalization for anxiety disorders was significantly more frequent in the youngest group (18–39) (Model 7: aOR = 2.44, 95%CI 1.07–5.56) and significantly less frequent in the oldest age groups (75+) (Model 7: aOR = 0.76, 95%CI 0.67–0.90) (Table 5).

**Table 5.** Adjusted odds ratio (OR) and 95% confidence interval (95%CI) for the risk of subsequent hospitalization for a personality disorder of different types over the 12-month period after initial hospital discharge, for patients hospitalized for COVID-19 versus those hospitalized for another reason, in all adult patients hospitalized in metropolitan France the first half of 2020: Model 7, stratified by age categories (18–39, 40–59, 60–74, and 75+) using fully-adjusted analysis.

	18–39	40–59	60–74	75 and more
	aOR (95%CI)^a^	aOR (95%CI)^a^	aOR (95%CI)^a^	aOR (95%CI)^a^
Any psychiatric disorder	0.818 (0.700–0.958)	0.939 (0.861–1.026)	1.097 (1.025–1.174)	0.935 (0.900–0.972)
Psychotic disorders	0.754 (0.521–1.091)	1.147 (0.940–1.401)	1.552 (1.295–1.861)	1.356 (0.954–1.830)
Anxiety disorders	2.442 (1.072–5.560)	0.889 (0.414–1.906)	1.412 (0.961–2.075)	0.775 (0.665–0.904)
Personality disorders	0.799 (0.585–1.090)	0.737 (0.588–0.923)	1.091 (0.897–1.328)	0.901 (0.780–1.040)

a *Odds-ratio* adjusted for socio-demographic characteristics (sex, region, and social deprivation index), psychiatric history and characteristics of the reference hospitalization: duration of hospitalization (days) and level of clinical care.

### Power analysis

All our models showed good power for the effect of initial COVID-19 hospitalization on subsequent hospitalization for a psychiatric disorder, even for small effect sizes. For example, the power curve for Model 4 for the primary outcome (most powerful model among all the Model 4 multivariable analyses performed [i.e., primary and secondary outcomes]) and the power curve for Model 6 for the secondary outcome ‘personality disorders’ (least powerful of all the models) are presented in the Supplementary Material (Supplementary Figures S2 and S3); other results are available on request.

## Discussion

To our knowledge, this is the first study to examine the risk of hospitalization for a psychiatric disorder during the 12 months following discharge after either hospitalization for COVID-19 or for another reason, using data from national administrative healthcare registers for the adult French general population. In unadjusted analyses, initial hospitalization for COVID-19 was associated with an approximately 20% higher risk of hospitalization for a psychiatric disorder (of any type) than initial hospitalization for another reason (11.09 vs. 9.24%, Model 1: OR = 1.20 95%CI 1.18–1.23). This difference highlights a serious public health issue, as hundreds of thousands of people have already been hospitalized for COVID-19 and thousands more will most likely be hospitalized in the future. In terms of specific disorders, this association was also observed for hospitalizations for psychotic disorders (1.37 vs. 0.90%, Model 1: OR = 1.11), mood disorders (1.05 vs. 0.95%, Model 1: OR = 1.12), and anxiety disorders (4.23 vs. 3.77%, Model 1: OR = 1.22).

After adjustment for sociodemographic data and psychiatric history, while initial hospitalization for COVID-19 remained significantly associated with the risk of hospitalization a psychiatric disorder of any type in the 12 months after the reference hospital stay, the association was weaker. This shows that the effect of hospitalization for COVID-19 on subsequent hospitalization can be partly explained by sex, age, and mostly by psychiatric history. Interestingly, adjustment for the level of clinical care received during the initial hospitalization reversed the direction of this association (from aOR = 1.06 to aOR = 0.93). As the level of clinical care can be considered an intermediate variable in the pathway between the reason of initial hospitalization, that is, COVID-19 versus another reason, and subsequent psychiatric hospitalization, the latter finding indicates that the effect of hospitalization not mediated by the level of clinical care was negative. In turn, initial hospitalization for COVID-19 in an ICU, and especially when associated with invasive ventilatory support, was positively associated with subsequent hospitalization for a psychiatric disorder (Model 4: aOR = 1.05 for patients in ICU or those who received noninvasive ventilatory support, and aOR = 1.16 for patients in ICU who received the most invasive type of ventilatory support). Likewise, as expected, psychiatric history was also associated with a higher risk of hospitalization for a psychiatric disorder (Model 3: aOR = 7.65 95%CI 7.58–7.72). Finally, while we found that psychiatric history was associated with subsequent psychiatric hospitalization in COVID-19 patients—which reflects findings elsewhere [26]—our stratified analyses revealed that this risk was especially high in individuals with no psychiatric history. That means that the risk of the onset of a new psychiatric disorder also increased. This reflects findings in other studies which found a risk of both first psychiatric diagnosis or relapse related to a previous diagnosis after COVID-19 infection [4, 11]. It is possible that patients with a relatively recent psychiatric history benefit from better monitoring and support, allowing them to avoid hospitalization for related disorders. Age-stratified analyses found a higher risk of subsequent hospitalization for a psychiatric disorder of any type (primary outcome) in the 60–74 age group (Model 7: aOR = 1.10, 95%CI 1.03–1.74), whereas the youngest (18–39) and oldest (75+) age groups had lower risk (Model 7: aOR = 0.82, 95%CI 0.70–0.96 and Model 7: aOR = 0.94, 95%CI 0.90–0.97, respectively).

Our results suggesting a higher risk of subsequent psychiatric hospitalization in patients hospitalized for COVID-19 than for another reason. For patients with the most severe forms of the disease, this could be due to the decrease in visits to outpatient psychiatric care and psychiatric emergency units during the early stage of pandemic to treat anxiety disorders, mood disorders and psychotic disorders [29–31]. Indeed, the decrease in the availability and/or the difficulty to use these support services (e.g., fear of contamination, fear of stigma, transport problems) result in delayed provision of care. In turn, this disruption leads to more severe disorders, increased treatment gaps, and other problems [32].

Several pathophysiological hypotheses can also be proposed to explain the association between COVID-19 and psychiatric disorders. COVID-19 has demonstrated neuroinvasive potential and neurotropism. Accordingly, following from the hypothesis of the involvement of inflammatory phenomena in psychiatric disorders etiology [33–35], direct and intense viral infiltration into the central nervous system may also play a role [36, 37]. Moreover, COVID-19 leads to a deregulation of cytokine networks (more acute in persons requiring intensive care) that can induce or decompensate psychiatric disorders [38, 39]. In addition, isolation and sensory deprivation associated with ICU stays are associated with psychiatric outcomes including anxiety, depressive and psychotic symptoms [40].

Psychological stressors associated with the pandemic (e.g., fear of the new disease, uncertainty about the future, general population concerns widely reported by the media, stigma), may also have increased the psychiatric impact of COVID-19 on ICU survivors [8,41–43].

The association between psychiatric history and hospitalization for psychiatric reasons within 12 months of initial hospitalization for COVID-19 may also be based on reverse causality. More specifically, the higher risk may not be related to COVID-19 hospitalization per se, but to selection bias: patients with a psychiatric history were at a higher risk of COVID-19 hospitalization, as a psychiatric history was the risk factor most strongly associated with subsequent hospitalization for a psychiatric disorder. Both hypotheses, that is, COVID-19 hospitalization as a risk factor for hospitalization for a psychiatric disorder, and vice-versa, may explain the association, and may not be mutually exclusive.

We found a significant risk of hospitalization for psychotic disorders over the 12 months following hospital discharge in patients initially hospitalized for COVID-19 than those initially hospitalized for another reason. This was true also after adjustment for socio-demographic characteristics and psychiatric history and it became slightly nonsignificant after adjustment for the characteristics of the reference hospitalization. This finding corroborates work conducted on previous epidemics and pandemics such as H1N1, Ebola, SARS, and MERS, which found a higher incidence of post-infection psychotic outcomes (disorders or symptoms) (0.9–4%) compared to the median incidence in the general population (0.015%) [44]. Case reports have also highlighted several acute psychotic episodes following COVID-19 infection [45, 46]. With regard to hospitalization for psychotic disorders, psychiatric history in our study was associated with a 30-fold higher risk of hospitalization. Elsewhere, patients suffering from schizophrenia had a higher risk of COVID-19 infection, and poorer prognosis following COVID-19, with more hospitalizations, higher mortality, and poorer quality follow-up over time than patients without severe psychiatric disorders [47, 48]. Interestingly, we found an association gradient between the level of clinical care received during hospitalization for COVID-19 and the risk of subsequent hospitalization for a psychotic disorder. This finding echoes results for SARS, where disease severity (and therefore the level of care) was an independent predictive factor, both at the acute stage of the infection and during convalescence [49].

Similarly, the risk of hospitalization for mood disorders appeared significantly higher in COVID-19 patients than in patients hospitalized for another medical reason (4.27 vs. 3.82%, Model 2: OR = 1.07 [1.03–1.1]). This risk was higher especially in women and elderly persons (75 years and over), reflecting findings elsewhere [8, 50, 51]. After adjustment for psychiatric history, this risk was no longer significantly higher (Model 3: OR = 0.97 [0.94–1]); this suggests that psychiatric history was even more strongly associated than sex and age with the risk of hospitalization for mood disorders. However this risk did not differ between patients hospitalized for COVID-19 or for another reason during the first 6 months of the pandemic. Few studies to date have focused on the impact of the COVID-19 pandemic on people with bipolar disorder [52]. When compared to people diagnosed with major depressive disorders, patients with bipolar disorder reported lower psychological distress but more severe cognitive symptoms, and greater pandemic-related stress, sleep disorders, and anxiety. It would therefore be interesting to address these disorders separately in future studies.

In the present study, hospitalizations for anxiety disorders appeared significantly more frequent in patients initially hospitalized for COVID-19 than those hospitalized for another reason, even after adjustment for socio-demographic characteristics and psychiatric history. Exposure to severe infection with respiratory symptoms is a traumatic event that can lead to increased experience of life-threat (56). Other studies have demonstrated the link between the severity of COVID-19 infection and the risk of anxiety disorders, in particular PTSD [53]. Specifically, in one study, PTSD was described in 20% of patients discharged 1 month earlier after hospitalization for the disease [54]. In our study, COVID-19 patients admitted to an ICU were more likely to be subsequently hospitalized for anxiety disorders (Model 4: OR 1.13 [1.09–1.17]). It has been established that the severity of respiratory symptoms, the fear of dying, and the fear of the unknown are all associated with the risk of PTSD [55, 56]. Furthermore, we showed that younger patients (under 40 years of age), hospitalized for COVID-19, had a higher risk of subsequent hospitalization for an anxiety disorder, whereas subjects over 75 years of age had a significantly lower risk of it. A recent meta-analysis also found that anxiety levels were consistently associated with younger age during the pandemic [57]. This could be explained by the relationship between resilience and anxiety among COVID-19 patients, with older subjects showing higher levels of resilience [58–60].

### Strengths and limitations

This study has several strengths. First, the SNDS healthcare database allowed us to conduct the first study to specifically investigate hospitalization for psychiatric disorders over the 12 months following discharge for hospitalization for COVID-19. Moreover, the SNDS provides detailed information for almost the entire French population [23], thereby limiting selection and memory biases, and ensuring analyses with good statistical power. Our analyses were conducted on a very large sample size (almost 3 million individuals), which is bigger than other studies that have focused until now on the same topic [17].

Second, the fact that hospitalizations for COVID-19 were compared to hospitalizations for a different reason during the same period is another major strength of this study, especially as the study period was marked by exceptional circumstances (strict lockdowns, stress, and fear of the infection). Third, the study period covered the 12 months after discharge from the initial hospitalization, which is longer than the majority of studies to date investigating the psychiatric sequelae of COVID-19 [61]. Finally, we not only studied psychiatric disorders globally, but also distinguished between them in our analyses.

There are also study limitations which should be considered when interpreting our results. We cannot know for sure if the pandemic and its related impact on hospital organizations during the first weeks may have impacted the quality of diagnosis coding. The process is carefully monitored by hospitals, since it is used for billing their services. However, it cannot be totally ruled out to this day.

In terms of access to intensive care, hospital management of COVID-19 in the first half of 2020 evolved according to bed availability. Accordingly, it is possible that our classification of the level of clinical care received did not accurately reflect the severity of the initial condition. For instance, the use of noninvasive ventilation outside of ICU was common for COVID-19 patients [62, 63]. In order to mitigate this bias, our analyses also took into account the ward type where specific care procedures were provided: for example, the second level of care corresponded to people in ICU but also those outside of ICU who received noninvasive procedures.

We were only able to look at psychiatric history in the 5 years previous to the study, meaning some people may have been misclassified as having no psychiatric history.

We did not evaluate the use of outpatient psychiatric care due to limitations in the SNDS chaining procedure linking nonhospital-based outpatient care with hospital-based care.

We did not have access to subsequent hospitalizations during the 12 months of follow-up for any reason other than psychiatric. This could have biased our results if another hospitalization related to the original reason for admission of the index hospital stay had occurred during the follow-up period. Further studies should specifically address this issue.

Finally, we analyzed categories of psychiatric disorders, and not disorders within each specific category (e.g., acute psychotic episode versus schizophrenia, or depressive disorder versus bipolar disorder). It is likely that individual disorders have different risks. It would be interesting to address this subject in further studies.

## Conclusion

During the first wave of the COIVD-19 pandemic in France, hospitalization for the disease compared with hospitalization for another reason, was associated with a higher risk of subsequent hospitalization for psychiatric disorders—both globally and for different specific psychiatric disorders—in the 12 months following discharge. This association was only marginally confounded by socio-demographics and having a psychiatric history. Furthermore, the association was reversed after adjusting for the level of care received and the duration of the initial hospital stay. Moreover, the strength of the association varied according to psychiatric history and the level of clinical care received. These findings should encourage clinicians to increase the monitoring and assessment of psychiatric symptoms after hospital discharge for COVID-19, and to propose post-hospital care, especially for those who have been treated in intensive care.

## Data Availability

The “Système national des données de santé” (SNDS, National French administrative healthcare database) comprises a set of strictly pseudonymized databases. By law, SpF has permanent regulatory access to SNDS data for the performance of its missions (article L.1461-3 and R1461-13 and following of the French public health code [28]). Access to personal data in these systems for research purposes is subject to obtaining an authorization from the *Commission nationale de l’informatique et des libertés* (CNIL, National Commission on Information Technology and Civil Liberties), after advice from the *Comité éthique et scientifique pour les recherches, les études et les évaluations dans le domaine de la santé* (CESREES, Ethics and Scientific Committee for Research, Studies and Evaluation in the field of health).

## Supplementary material

For supplementary material accompanying this paper visit https://doi.org/10.1192/j.eurpsy.2022.2331. click here to view supplementary material
