# Supplementary material for: Hospitalization for COVID-19 is associated with a higher risk of subsequent hospitalization for psychiatric disorders: A French nationwide longitudinal study comparing hospitalizations for COVID-19 and for other reasons
Source: Eur Psychiatry. 2022 Oct 21;65(1):e70. doi: 10.1192/j.eurpsy.2022.2331 (PMC9677447; doi:10.1192/j.eurpsy.2022.2331)
Supplement: Supplementary file 1 [file S0924933822023318sup001.docx]

| *Second level : patients admitted to an intensive care unit (ICU), irrespective of the the level and type of oxygen supply therapy, and patients who received high flow nasal oxygen or non-invasive ventilation* | |
| --- | --- |
| GLLD003 | Spontaneous ventilation with face mask, nasal cannula or nasopharyngeal tube, with no inspiratory support, with positive expiratory pressure [VS-PEP] [Continuous positive airway pressure] [CPAP], for 24 hours |
| GLLD012 | Continuous mechanical ventilation with face mask for ventilatory support, for 24 hours |
| GLLD019 | Non-invasive barometric or volume ventilation with face mask for at least 2 hours (cumulative) over 12 hours for acute respiratory failure |
| GLLD017 | Oxygen therapy with continuous oximetry monitoring, outside of mechanical ventilation, for 24 hours |
| GLQF001 | Adjustment of oxygen flow by repeated blood gas measurements, for initiation or adaptation of defined flow oxygen therapy, for 24 hours |
| *Maximum severity level: patients admitted to an ICU who required at least invasive ventilatory support* | |
| DKMD001 | Cardiopulmonary resuscitation with tracheal intubation outside of a medical-technical block |
| DKMD002 | Cardiopulmonary resuscitation with tracheal intubation inside a medical-technical block |
| EQQP004 | Hemodynamic support by extracorporeal circulation, for 24 hours |
| GLJF010 | Extracorporeal carbon dioxide [CO2] removal, for 24 hours |
| GLLD004 | Intratracheal mechanical ventilation with positive end expiratory pressure [PEEP] greater than 6 and/or FiO2 greater than 60%, with alternating prone technique for 24 hours |
| GLLD007 | Mechanical ventilation with separate lungs, for 24 hours |
| GLLD008 | Intratracheal mechanical ventilation with positive end expiratory pressure [PEEP] greater than 6 and/or FiO2 greater than 60%, for 24 hours |
| GLLD009 | High frequency oscillation ventilation, for 24 hours |
| GLLD015 | Intratracheal mechanical ventilation with positive expiratory pressure [PEEP] less than or equal to 6 and FiO2 less than or equal to 60%, for 24 hours |
| GLQP015 | Continuous monitoring of jugular oxygen saturation by intravenous device, for 24 hours |
| INTUBATION | GELD004 GELE004 GELE001 GELD002 |
| TRACHEOTOMIE | GEPA004 |
| EQLA002 | Placement of an emergency extracorporeal circulation for circulatory assistance, by peripheral vascular approach |
| ZZLB004 | Continuous sedation and curarization, with monitoring of curarization by spinal nerve stimulator, for 24 hours |

**Supplementary materials**

**Table S1.** List of procedures used to define the second and maximum evels of clinical care

**Table S2.** Comparison of reasons for hospitalization for a psychiatric disorder in patients previously hospitalized for COVID-19 vs for another reason (percentages of total hospitalizations for each category of psychiatric disorder).

|  | Total (n= 2,894,088) | Hospitalizations for COVID-19 (n= 96,313) | Hospitalizations for any other reason (n= 2,979,775 ) | *P-*value |
| --- | --- | --- | --- | --- |
| *Reason for hospitalization* |  |  |  |  |
| All psychiatric disorders | 269,251 (9.3) | 10,685 (11.09) | 258,566 (9.24) | <.0001 |
| Psychotic disorders | 27,614 (0.95) | 1,019 (1.06) | 26,595 (0.95) | 0.0007 |
| Mood disorders | 110,976 (3.83) | 4,115 (4.27) | 106,861 (3.82) | <.0001 |
| Anxiety disorders | 146,653 (5.07) | 5,834 (6.06) | 140,819 (5.03) | <.0001 |
| Behavioral syndromes | 10,786 (0.37) | 370 (0.38) | 10,416 (0.37) | 0.55 |
| Personality disorders | 27,948 (0.97) | 766 (0.8) | 27,182 (0.97) | <.0001 |

|  | Model 1 * | | Model 2 ** | | Model 3 *** | | Model 4 **** | |
| --- | --- | --- | --- | --- | --- | --- | --- | --- |
|  | OR (95%CI) | *P-*value | aOR (95%CI) | *P-*value | aOR (95%CI) | *P-*value | aOR (95%CI) | *P-*value |
| *Hospitalization for COVID-19* |  |  |  |  |  |  |  |  |
| No | 1 |  | 1 |  | 1 |  | 1 |  |
| Yes | 1.11 [1.05-1.19] | <.0001 | 1.16 [1.09-1.24] | <.0001 | 1.09 [1.02-1.17] | 0.0093 | 1.06 [0.99-1.14] | 0.09 |
| *Sex* |  |  |  |  |  |  |  |  |
| Male |  |  | 1 |  | 1 |  | 1 |  |
| Female |  |  | 0.65 [0.63-0.67] | <.0001 | 0.58 [0.56-0.59] | <.0001 | 0.58 [0.56-0.59] | <.0001 |
| *Age (years)* |  |  |  |  |  |  |  |  |
| 18-39 |  |  | 1 |  | 1 |  | 1 |  |
| 40-59 |  |  | 1.15 [1.12-1.19] | <.0001 | 0.68 [0.66-0.7] | <.0001 | 0.68 [0.65-0.7] | <.0001 |
| 60-74 |  |  | 0.65 [0.62-0.67] | <.0001 | 0.47 [0.45-0.49] | <.0001 | 0.47 [0.45-0.48] | <.0001 |
| 75+ |  |  | 0.44 [0.43-0.46] | <.0001 | 0.28 [0.27-0.29] | <.0001 | 0.28 [0.27-0.29] | <.0001 |
| *Social deprivation index (quintiles)* |  |  |  |  |  |  |  |  |
| 1 (least deprived) |  |  | 1 |  | 1 |  | 1 |  |
| 2 |  |  | 0.91 [0.87-0.95] | <.0001 | 0.92 [0.88-0.96] | 0.0003 | 0.92 [0.88-0.96] | 0.0003 |
| 3 |  |  | 1.08 [1.04-1.13] | 0.0001 | 1.02 [0.98-1.07] | 0.282 | 1.02 [0.98-1.07] | 0.3064 |
| 4 |  |  | 1.01 [0.97-1.05] | 0.4816 | 0.95 [0.91-0.99] | 0.0163 | 0.95 [0.91-0.99] | 0.013 |
| 5 (most deprived) |  |  | 1.05 [1-1.09] | 0.0444 | 0.97 [0.93-1.01] | 0.1465 | 0.97 [0.93-1.01] | 0.1239 |
| *Psychiatric history* |  |  |  |  |  |  |  |  |
| No |  |  |  |  | 1 |  | 1 |  |
| Yes |  |  |  |  | 33.28 [32.32-34.26] | <.0001 | 33.16 [32.21-34.14] | <.0001 |
| *Duration of reference hospitalization (days) - mean (SD)* |  |  |  |  |  |  | 1 [1-1.01] | <.0001 |
| *Level of clinical care* |  |  |  |  |  |  |  |  |
| General hospital ward (medical, surgery, obstetrics) |  |  |  |  |  |  | 1 |  |
| Intensive care unit |  |  |  |  |  |  | 0.87 [0.85-0.93] | <.0001 |
| Intensive care unit with invasive  procedures |  |  |  |  |  |  | 1.24 [1.15-1.33] | <.0001 |

**Table S3.** Odds-ratio (OR), adjusted odds-ratio (aOR) and 95% confidence interval (95%CI) for the risk of subsequent hospitalization for psychotic disorders over the 12-month period after initial hospital discharge, for patients hospitalized for COVID-19 versus those hospitalization for another reason, in all adult patients hospitalized in metropolitan France the first half of 2020.

| * No adjustment |
| --- |
| ** *Odds ratio* adjusted for socio-demographic characteristics : sex, age, region and social deprivation index |
| *** *Odds ratio* adjusted for socio-demographic characteristics and psychiatric history |
| **** *Odds ratio* adjusted for socio-demographic characteristics, psychiatric history and characteristics of the reference hospitalization : duration of hospitalization (days) and level of clinical care |

|  | Model 1 * | | Model 2 ** | | Model 3 *** | | Model 4 **** | |
| --- | --- | --- | --- | --- | --- | --- | --- | --- |
|  | OR (95%CI) | *P-*value | aOR (95%CI) | *P-*value | aOR (95%CI) | *P-*value | aOR (95%CI) | *P-*value |
| *Hospitalization for COVID-19* |  |  |  |  |  |  |  |  |
| No | 1 |  | 1 |  | 1 |  | 1 |  |
| Yes | 1.12 [1.09-1.16] | <.0001 | 1.07 [1.03-1.1] | 0.0001 | 0.97 [0.94-1] | 0.0778 | 0.87 [0.84-0.9] | <.0001 |
| *Sex* |  |  |  |  |  |  |  |  |
| Male |  |  | 1 |  | 1 |  | 1 |  |
| Female |  |  | 1.35 [1.34-1.37] | <.0001 | 1.15 [1.14-1.17] | <.0001 | 1.17 [1.15-1.18] | <.0001 |
| *Age (years)* |  |  |  |  |  |  |  |  |
| 18-39 |  |  | 1 |  | 1 |  | 1 |  |
| 40-59 |  |  | 2.44 [2.39-2.49] | <.0001 | 1.75 [1.71-1.79] | <.0001 | 1.72 [1.68-1.75] | <.0001 |
| 60-74 |  |  | 1.97 [1.93-2.01] | <.0001 | 1.56 [1.53-1.6] | <.0001 | 1.49 [1.45-1.52] | <.0001 |
| 75+ |  |  | 2.22 [2.17-2.26] | <.0001 | 1.65 [1.62-1.68] | <.0001 | 1.55 [1.52-1.58] | <.0001 |
| *Social deprivation index (quintiles)* |  |  |  |  |  |  |  |  |
| 1 (least deprived) |  |  | 1 |  | 1 |  | 1 | 1 |
| 2 |  |  | 0.92 [0.9-0.94] | <.0001 | 0.93 [0.91-0.95] | <.0001 | 0.93 [0.91-0.95] | <.0001 |
| 3 |  |  | 0.96 [0.94-0.98] | <.0001 | 0.92 [0.9-0.94] | <.0001 | 0.92 [0.9-0.95] | <.0001 |
| 4 |  |  | 0.94 [0.92-0.96] | <.0001 | 0.9 [0.88-0.92] | <.0001 | 0.9 [0.88-0.92] | <.0001 |
| 5 (most deprived) |  |  | 0.96 [0.94-0.98] | <.0001 | 0.92 [0.9-0.94] | <.0001 | 0.91 [0.89-0.93] | <.0001 |
| *Psychiatric history* |  |  |  |  |  |  |  |  |
| No |  |  |  |  | 1 |  | 1 |  |
| Yes |  |  |  |  | 9.69 [9,56-9,81] | <.0001 | 9.65 [9.53-9.77] | <.0001 |
| *Duration of reference hospitalization (days) – mean (SD)* |  |  |  |  |  |  | 1.02 [1.01-1.03] | <.0001 |
| *Level of clinical care* |  |  |  |  |  |  |  |  |
| General hospital ward (medical, surgery, obstetrics) |  |  |  |  |  |  | 1 |  |
| Intensive care unit |  |  |  |  |  |  | 1.07 [1.05-1.10] | <.0001 |
| Intensive care unit with invasive  procedures |  |  |  |  |  |  | 1.3 [1.25-1.35] | <.0001 |

**Table S4.**  Odds-ratio (OR), adjusted odds-ratio (aOR) and 95% confidence interval (95%CI) for the risk of subsequent hospitalization for mood disorders over the 12-month period after initial hospital discharge, for patients hospitalized for COVID-19 versus those hospitalization for another reason, in all adult patients hospitalized in metropolitan France the first half of 2020.

| * No adjustment |
| --- |
| ** *Odds ratio* adjusted for socio-demographic characteristics : sex, age, region and social deprivation index |
| *** *Odds ratio* adjusted for socio-demographic characteristics and psychiatric history |
| **** *Odds ratio* adjusted for socio-demographic characteristics, psychiatric history and characteristics of the reference hospitalization : duration of hospitalization (days) and level of clinical care |

|  | Model 1 * | | Model 2 ** | | Model 3 *** | | Model 4 **** | |
| --- | --- | --- | --- | --- | --- | --- | --- | --- |
|  | OR (95%CI) | *P* value | aOR (95%CI) | *P* value | aOR (95%CI) | *P* value | aOR (95%CI) | *P* value |
| *Hospitalization for COVID-19* |  |  |  |  |  |  |  |  |
| No | 1 |  | 1 |  | 1 |  | 1 |  |
| Yes | 1.22 [1.18-1.25] | <.0001 | 1.18 [1.14-1.21] | <.0001 | 1.11 [1.08-1.14] | <.0001 | 0.98 [0.95-1.01] | 0.1536 |
| *Sex* |  |  |  |  |  |  |  |  |
| Male |  |  | 1 |  | 1 |  | 1 | 1 |
| Female |  |  | 1.4 [1.38-1.41] | <.0001 | 1.26 [1.25-1.28] | <.0001 | 1.28 [1.27-1.29] | <.0001 |
| *Age* |  |  |  |  |  |  |  |  |
| 18-39 |  |  | 1 |  | 1 |  | 1 |  |
| 40-59 |  |  | 2.12 [2.08-2.16] | <.0001 | 1.75 [1.72-1.79] | <.0001 | 1.71 [1.68-1.75] | <.0001 |
| 60-74 |  |  | 2.04 [2-2.08] | <.0001 | 1.79 [1.76-1.83] | <.0001 | 1.69 [1.66-1.72] | <.0001 |
| 75+ |  |  | 3.12 [3.07-3.17] | <.0001 | 2.68 [2.63-2.73] | <.0001 | 2.47 [2.42-2.51] | <.0001 |
| *Social deprivation index (quintiles)* |  |  |  |  |  |  |  |  |
| 1 (least deprived) |  |  | 1 |  | 1 |  | 1 |  |
| 2 |  |  | 0.97 [0.96-0.99] | 0.0041 | 0.98 [0.96-1] | 0.0253 | 0.98 [0.96-1] | 0.0101 |
| 3 |  |  | 0.99 [0.98-1.01] | 0.2843 | 0.97 [0.96-0.99] | 0.0084 | 0.97 [0.95-0.99] | 0.0009 |
| 4 |  |  | 1.02 [1-1.04] | 0.0488 | 1 [0.98-1.02] | 0.8311 | 0.99 [0.97-1.01] | 0.4761 |
| 5 (most deprived) |  |  | 1.02 [1-1.04] | 0.0158 | 1 [0.98-1.02] | 0.7562 | 0.99 [0.97-1.01] | 0.3743 |
| *Psychiatric history* |  |  |  |  |  |  |  |  |
| No |  |  |  |  | 1 |  | 1 |  |
| Yes |  |  |  |  | 4.33 [4.28-4.38] | <.0001 | 4.31 [4.26-4.36] | <.0001 |
| *Duration of reference hospitalization (days) – mean (SD)* |  |  |  |  |  |  | 1.02 [1.02-1.02] | <.0001 |
| *Level of clinical care* |  |  |  |  |  |  |  |  |
| General hospital ward (medical, surgery, obstetrics) |  |  |  |  |  |  | 1 |  |
| Intensive care unit |  |  |  |  |  |  | 1.07 [1.05-1.09] | <.0001 |
| Intensive care unit with invasive  procedures |  |  |  |  |  |  | 1.13 [1.09-1.17] | <.0001 |

**Table S5.** Odds-ratio (OR), adjusted odds-ratio (aOR) and 95% confidence interval (95%CI) for the risk of subsequent hospitalization for anxiety disorders over the 12-month period after initial hospital discharge, for patients hospitalized for COVID-19 versus those hospitalization for another reason, in all adult patients hospitalized in metropolitan France the first half of 2020.

| * No adjustment |
| --- |
| ** *Odds ratio* adjusted for socio-demographic characteristics : sex, age, region and social deprivation index |
| *** *Odds ratio* adjusted for socio-demographic characteristics and psychiatric history |
| **** *Odds ratio* adjusted for socio-demographic characteristics, psychiatric history and characteristics of the reference hospitalization : duration of hospitalization (days) and level of clinical care |

|  | Model 1 * | | Model 2 ** | | Model 3 *** | | Model 4 **** | |
| --- | --- | --- | --- | --- | --- | --- | --- | --- |
|  | OR (95%CI) | *P* value | aOR (95%CI) | *P* value | aOR (95%CI) | *P* value | aOR (95%CI) | *P* value |
| *Hospitalization for COVID-19* |  |  |  |  |  |  |  |  |
| No | 1 |  | 1 |  | 1 |  | 1 |  |
| Yes | 0.82 [0.76-0.88] | <.0001 | 0.92 [0.86-0.99] | 0.0319 | 0.86 [0.8-0.92] | 0.0001 | 0.82 [0.76-0.88] | <.0001 |
| *Sex* |  |  |  |  |  |  |  |  |
| Male |  |  | 1 |  | 1 |  | 1 |  |
| Female |  |  | 0.74 [0.72-0.76] | <.0001 | 0.68 [0.66-0.7] | <.0001 | 0.68 [0.66-0.7] | <.0001 |
| *Age (years)* |  |  |  |  |  |  |  |  |
| 18-39 |  |  | 1 |  | 1 |  | 1 |  |
| 40-59 |  |  | 0.99 [0.96-1.03] | 0.7103 | 0.68 [0.65-0.7] | <.0001 | 0.67 [0.65-0.69] | <.0001 |
| 60-74 |  |  | 0.42 [0.4-0.43] | <.0001 | 0.33 [0.32-0.34] | <.0001 | 0.32 [0.31-0.34] | <.0001 |
| 75+ |  |  | 0.55 [0.53-0.56] | <.0001 | 0.4 [0.38-0.41] | <.0001 | 0.39 [0.38-0.4] | <.0001 |
| *Social deprivation index (quintiles)* |  |  |  |  |  |  |  |  |
| 1 (least deprived) |  |  | 1 |  | 1 |  | 1 |  |
| 2 |  |  | 0.9 [0.86-0.94] | <.0001 | 0.91 [0.87-0.95] | <.0001 | 0.91 [0.87-0.95] | <.0001 |
| 3 |  |  | 0.97 [0.94-1.02] | 0.2682 | 0.93 [0.9-0.97] | 0.0015 | 0.93 [0.89-0.97] | 0.0012 |
| 4 |  |  | 0.99 [0.95-1.03] | 0.5824 | 0.94 [0.9-0.98] | 0.0039 | 0.43 [0.9-0.98] | 0.0027 |
| 5 (most deprived) |  |  | 0.97 [0.93-1.01] | 0.1982 | 0.91 [0.88-0.95] | <.0001 | 0.91 [0.87-0.95] | <.0001 |
| *Psychiatric history* |  |  |  |  |  |  |  |  |
| No |  |  |  |  | 1 |  | 1 |  |
| Yes |  |  |  |  | 13.5 [13.17-13.84] | <.0001 | 13.45 [13.12-13.78 | <.0001 |
| *Duration of reference hospitalization (days) – mean (SD)* |  |  |  |  |  |  | 1.01 [1.01-1.01] | <.0001 |
| *Level of clinical care* |  |  |  |  |  |  |  |  |
| General hospital ward (medical, surgery, obstetrics) |  |  |  |  |  |  | 1 |  |
| Intensive care unit |  |  |  |  |  |  | 0.96 [0.92-1] | 0.0602 |
| Intensive care unit with invasive  procedures |  |  |  |  |  |  | 1.24 [1.15-1.33] | <.0001 |

**Table S6.** Odds-ratio (OR), adjusted odds-ratio (aOR) and 95% confidence interval (95%CI) for the risk of subsequent hospitalization for personality disorders over the 12-month period after initial hospital discharge, for patients hospitalized for COVID-19 versus those hospitalization for another reason, in all adult patients hospitalized in metropolitan France the first half of 2020.

| * No adjustment |
| --- |
| ** *Odds ratio* adjusted for socio-demographic characteristics : sex, age, region and social deprivation index |
| *** *Odds ratio* adjusted for socio-demographic characteristics and psychiatric history |
| **** *Odds ratio* adjusted for socio-demographic characteristics, psychiatric history and characteristics of the reference hospitalization : duration of hospitalization (days) and level of clinical care |

**Table S7.** Adjusted Odds ratio (OR) and 95% confidence interval (95%CI) for the risk of subsequent hospitalization for psychotic disorders over the 12-month period after initial hospital discharge, for patients hospitalized for COVID-19 versus those hospitalization for another reason, in all adult patients hospitalized in metropolitan France the first half of 2020: Model 5, stratified by a psychiatric history vs no psychiatric history using the model adjusted for all variables (Model 4)

|  | Psychiatric history | | No psychiatric history | |
| --- | --- | --- | --- | --- |
|  | aOR (95%CI) | *P* value | aOR (95%CI) | *P* value |
| *Hospitalization with COVID-19* |  |  |  |  |
| No | 1 |  | 1 |  |
| Yes | 0.82 [0.78-0.87] | <.0001 | 0.98 [0.93-1.02] | 0.1427 |
| *Sex* |  |  |  |  |
| Male | 1 |  | 1 |  |
| Female | 1.23 [1.2-1.25] | <.0001 | 1.23 [1.21-1.25] | <.0001 |
| *Age (years)* |  |  |  |  |
| 18-39 | 1 |  | 1 |  |
| 40-59 | 1.22 [1.19-1.26] | <.0001 | 2.13 [2.07-2.2] | <.0001 |
| 60-74 | 1.03 [1-1.06] | 0.0811 | 2 [1.94-2.07] | <.0001 |
| 75+ | 0.65 [0.63-0.67] | <.0001 | 3.06 [2.97-3.15] | <.0001 |
| *Social deprivation index (quintiles)* |  |  |  |  |
| 1 (least deprived) | 1 |  | 1 |  |
| 2 | 0.93 [0.9-0.96] | <.0001 | 0.92 [0.9-0.95] | <.0001 |
| 3 | 0.9 [0.87-0.93] | <.0001 | 0.92 [0.9-0.95] | <.0001 |
| 4 | 0.84 [0.82-0.87] | <.0001 | 0.93 [0.91-0.96] | <.0001 |
| 5 (most deprived) | 0.86 [0.83-0.88] | <.0001 | 0.94 [0.91-0.97] | <.0001 |
| *Duration of reference hospitalization (days) - mean (SD)* | 1 [1-1.01] | <.0001 | 1,02 [1.02-1.02] | <.0001 |
| *Level of clinical care* |  |  |  |  |
| General hospital ward (medical, surgery, obstetrics) | 1 |  | 1 |  |
| Intensive care unit | 1.08 [1.05] | <.0001 | 1.01 [0.99-1.04] | 0.3537 |
| Intensive care unit with invasive  procedures | 1.26 [1.19-1.33] | <.0001 | 1.21 [1.15-1.27] | <.0001 |

| *Odds ratio* adjusted for socio-demographic characteristics and characteristics of the reference hospitalization |
| --- |

|  | Psychiatric history | | No psychiatric history | |  | General hospital wards | | | Intensive care unit | | Intensive care unit with invasive procedures | |
| --- | --- | --- | --- | --- | --- | --- | --- | --- | --- | --- | --- | --- |
|  | aOR (95%CI) | *P* value | aOR (95%CI) | *P* value |  | aOR (95%CI) | *P* value | | aOR (95%CI) | *P* value | aOR (95%CI) | *P* value |
| *Hospitalization with COVID-19* |  |  |  |  |  |  | |  |  |  |  |  |
| No | 1 |  | 1 |  |  | 1 | |  | 1 |  | 1 |  |
| Yes | 0.85 [0.81-0.9] | <.0001 | 1.07 [1,04-1.11] | 0.001 |  | 0.94 [0.91-0.97] | | <.0001 | 0.92 [0.85-0.99] | 0.036 | 1.36 [1.24-1.48] | <.0001 |
| *Sex* |  |  |  |  |  |  | |  |  |  |  |  |
| Male | 1 |  | 1 |  |  | 1 | |  | 1 |  | 1 |  |
| Female | 1.25 [1.23-1.28] | <.0001 | 1.37 [1.35-1.39] | <.0001 |  | 1.24 [1.22-1.25] | | <.0001 | 1.58 [1.53-1.63] | <.0001 | 1.63 [1.53-1.74] | <.0001 |
| *Age (years)* |  |  |  |  |  |  | |  |  |  |  |  |
| 18-39 | 1 |  | 1 |  |  | 1 | |  | 1 |  | 1 |  |
| 40-59 | 0.96 [0.93-0.99] | 0.0209 | 2.15 [2.09-2.2] | <.0001 |  | 1.77 [1.73-1.81] | | <.0001 | 1.02 [0.95-1.09] | 0.611 | 0.76 [0.68-0.84] | <.0001 |
| 60-74 | 0.83 [0.8-0.86] | <.0001 | 2.34 [2.28-2.4] | <.0001 |  | 1.76 [1.73-1.8] | | <.0001 | 0.93 [0.88-0.99] | 0.0286 | 0.6 [0.54-0.67] | <.0001 |
| 75+ | 0.89 [0.87-0.92] | <.0001 | 3.82 [3.74-3.91] | <.0001 |  | 2.66 [2.61-2.71] | | <.0001 | 1.11 [1.05-1.19] | 0.0006 | 0.5 [0.44-0.56] | <.0001 |
| *Social deprivation index (quintiles)* |  |  |  |  |  |  | |  |  |  |  |  |
| 1 (least deprived) | 1 |  | 1 |  |  | 1 | |  | 1 |  | 1 |  |
| 2 | 1.01 [0.97-1.04] | 0.7531 | 0.96 [0.94-0.98] | 0.0002 |  | 0.97 [0.95-0.95-0.99] | | 0.0079 | 0.97 [0.92-1.02] | 0.2419 | 1.04 [0.93-1.15] | 0.517 |
| 3 | 0.94 [0.91-0.98] | 0.0011 | 0.97 [0.95-0.99] | 0.0131 |  | 0.97 [0.95-0.99] | | 0.0008 | 0.98 [0.92-1.03] | 0.412 | 0.94 [0.85-1.05] | 0.294 |
| 4 | 0.97 [0.94-1] | 0.0785 | 1 [0.97-1.02] | 0.6792 |  | 0.99 [0.97-1.01] | | 0.5174 | 0.98 [0.93-1.04] | 0.4713 | 0.93 [0.83-1.03] | 0.1586 |
| 5 (most deprived) | 0.95 [0.92-0.98] | 0.0033 | 1 [0.98-1.02] | 0.9211 |  | 0.99 [0.97-1.01] | | 0.371 | 0.97 [0.92-1.03] | 0.2699 | 0.98 [0.88-1.09] | 0.6479 |
| *History of psychiatric care* |  |  |  |  |  |  | |  |  |  |  |  |
| No |  |  |  |  |  | 1 | |  | 1 |  | 1 |  |
| Yes |  |  |  |  |  | 4.33 [4.28-4.39] | | <.0001 | 3.74 [3.61-3.88] | <.0001 | 3.11 [2.9-3.35] | <.0001 |
| *Duration of reference hospitalization (days) - mean (SD)* | 1.01 [1.01-1.01] | <.0001 | 1.02 [1.02-1.02] | <.0001 |  | 1.02 [1.02-1.02] | | <.0001 | 1.02 [1.02-1.02] | <.0001 | 1.01 [1.01-1.01] | <.0001 |
| *Level of clinical care* |  |  |  |  |  |  | |  |  |  |  |  |
| General hospital ward (medical, surgery, obstetrics) | 1 |  | 1 |  |  |  | |  |  |  |  |  |
| Intensive care unit | 1.05 [1.01-1.08] | 0.0034 | 1.04 [1.02-1.07] | <.0001 |  |  | |  |  |  |  |  |
| Intensive care unit with invasive  procedures | 1.07 [1-1.14] | 0.0333 | 1.08 [1.04-1.12] | 0.0002 |  |  | |  |  |  |  |  |

**Table S8.** Adjusted Odds ratio (OR) and 95% confidence interval (95%CI) for the risk of subsequent hospitalization for anxiety disorders over the 12-month period after initial hospital discharge, for patients hospitalized for COVID-19 versus those hospitalization for another reason, in all adult patients hospitalized in metropolitan France the first half of 2020: Model 5, stratified by psychiatric history vs. no psychiatric history using fully-adjusted analysis (Model 4), and Model 6, stratified by level of clinical care using model adjusted for all variables (Model 4).

|  | Psychiatric history | | No psychiatric history | |  | General hospital wards | | | Intensive care unit | | Intensive care unit with invasive procedures | |
| --- | --- | --- | --- | --- | --- | --- | --- | --- | --- | --- | --- | --- |
|  | aOR (95%CI) | *P* value | aOR (95%CI) | *P* value |  | aOR (95%CI) | *P* value | | aOR (95%CI) | *P* value | aOR (95%CI) | *P* value |
| *Hospitalization with COVID-19* |  |  |  |  |  |  | |  |  |  |  |  |
| No | 1 |  | 1 |  |  | 1 | |  | 1 |  | 1 |  |
| Yes | 0.83 [0.75-0.97] | 0.0008 | 0.9 [0.81-1] | 0.0526 |  | 0.86 [0.8-0.94] | | 0.0004 | 0.86 [0.7-1.06] | 0.2258 | 0.32 [0.22-0.48] | <.0001 |
| *Sex* |  |  |  |  |  |  | |  |  |  |  |  |
| Male | 1 |  | 1 |  |  | 1 | |  | 1 |  | 1 |  |
| Female | 0.88 [0.85-0.9] | <.0001 | 0.62 [0.59-0.64] | <.0001 |  | 0.65 [0.63-0.67] | | <.0001 | 0.96 [0.88-1.04] | 0.3073 | 1.44 [1.25-1.66] | <.0001 |
| *Age (years)* |  |  |  |  |  |  | |  |  |  |  |  |
| 18-39 | 1 |  | 1 |  |  | 1 | |  | 1 |  | 1 |  |
| 40-59 | 0.51 [0.49-0.53] | <.0001 | 0.9 [0.85-0.96] | 0.0005 |  | 0.71 [0.69-0.74] | | <.0001 | 0.37 [0.33-0.41] | <.0001 | 0.34 [0.29-0.4] | <.0001 |
| 60-74 | 0.22 [0.21-0.23] | <.0001 | 0.55 [0.52-0.58] | <.0001 |  | 0.35 [0.33-0.36] | | <.0001 | 0.17 [0.15-0.19] | <.0001 | 0.14 [0.11-0.17] | <.0001 |
| 75+ | 0.13 [0.12-0.13] | <.0001 | 1.17 [1.12-1.23] | <.0001 |  | 0.42 [0.41-0.44] | | <.0001 | 0.17 [0.15-0.19] | <.0001 | 0.07 [0.05-0.1] | <.0001 |
| *Social deprivation index (quintiles)* |  |  |  |  |  |  | |  |  |  |  |  |
| 1 (least deprived) | 1 |  | 1 |  |  | 1 | |  | 1 |  | 1 |  |
| 2 | 0.92 [0.87-0.98] | 0.0066 | 0.89 [0.83-0.95] | 0.0002 |  | 0.9 [0.86-0.94] | | <.0001 | 1.01 [0.88-0.16] | 0.8657 | 0.91 [0.72-0.17] | 0.4633 |
| 3 | 0.95 [0.9-1] | 0.0583 | 0.89 [0.84-0.95] | 0.0006 |  | 0.93 [0.89-0.98] | | 0.0028 | 0.96 [0.83-1.1] | 0.557 | 0.78 [0.61-1] | 0.0487 |
| 4 | 0.92 [0.87-0.97] | 0.0027 | 0.95 [0.89-1] | 0.0864 |  | 0.94 [0.9-0.98] | | 0.0037 | 1.02 [0.89-1.18] | 0.7377 | 0.73 [0.57-0.94] | 0.0136 |
| 5 (most deprived) | 0.86 [0.81-0.91] | <.0001 | 0.96 [0.9-1.02] | 0.1721 |  | 0.92 [0.88-0.96] | | 0.0002 | 0.91 [0.79-1.05] | 0.1824 | 0,8 [0.62-1.02] | 0.0752 |
| *History of psychiatric care* |  |  |  |  |  |  | |  |  |  |  |  |
| No |  |  |  |  |  | 1 | |  | 1 |  | 1 |  |
| Yes |  |  |  |  |  | 13.18 [12.84-13.54] | | <.0001 | 11.95 [11-12.98] | <.0001 | 11.86 [10.19-13.81] | <.0001 |
| *Duration of reference hospitalization (days) - mean (SD)* | 0.99 [0.99-1] | <.0001 | 1.02 [1.02-1.02] | <.0001 |  | 1.01 [1-1.01] | | <.0001 | 1.01 [1-1.01] | 0.0014 | 0.99 [0.99-1] | 0.0027 |
| *Level of clinical care* |  |  |  |  |  |  | |  |  |  |  |  |
| General hospital ward (medical, surgery, obstetrics) | 1 |  | 1 |  |  |  | |  |  |  |  |  |
| Intensive care unit | 1.04 [0.98-1.10] | 0.2763 | 0.8 [0.75-0.85] | <.0001 |  |  | |  |  |  |  |  |
| Intensive care unit with invasive  procedures | 1.46 [1.33-1.6] | <.0001 | 0.81 [0.71-0.92] | <.0001 |  |  | |  |  |  |  |  |

**Table S9.** Adjusted Odds ratio (OR) and 95% confidence interval (95%CI) for the risk of subsequent hospitalization for personality disorders over the 12-month period after initial hospital discharge, for patients hospitalized for COVID-19 versus those hospitalization for another reason, in all adult patients hospitalized in metropolitan France the first half of 2020: Model 5, stratified by psychiatric history vs. no psychiatric history using fully-adjusted analysis (Model 4), and Model 6, stratified by level of clinical care using model adjusted for all variables (Model 4)

| **Table S10.**  Adjusted Odds ratio (OR) and 95% confidence interval (95%CI) for the risk of subsequent hospitalization for a psychiatric disorder of any type over the 12-month period after initial hospital discharge, for patients hospitalized for COVID-19 versus those hospitalized for another reason, in all adult patients hospitalized in metropolitan France the first half of 2020: Model 7, stratified by age categories (18-39, 40-59, 60-74, and 75+) using fully-adjusted analysis (Model 4) | | | | | | | | | | | |
| --- | --- | --- | --- | --- | --- | --- | --- | --- | --- | --- | --- |
|  | 18-39 | | |  | 40-59 | |  | 60-74 | | 75 and more | |
|  | aOR (95%CI) | | *P* value |  | aOR (95%CI) | *P* value |  | aOR (95%CI) | *P* value | aOR (95%CI) | *P* value |
| *Hospitalization for COVID-19* | |  |  |  |  |  |  |  |  |  |  |
| No | | 1 |  |  | 1 |  |  | 1 |  | 1 |  |
| Yes | | **0.818 [0.700-0.958]** | **0.0124** |  | 0.939 [0.861-1.026] | 0.1629 |  | **1.097 [1.025-1.174]** | **0,0074** | **0.935 [0.900-0.972]** | **0,006** |
| *Sex* | |  |  |  |  |  |  |  |  |  |  |
| Male | | 1 |  |  | 1 |  |  | 1 |  | 1 |  |
| Female | | **0.522 [0.504-0.540]** | **<.0001** |  | **0.873 [0.846-0.900]** | **<.0001** |  | **0.907 [0.881-0.933]** | **<.0001** | **1.052 [1.035-1.070]** | **<.0001** |
| *Social deprivation index (quintiles)* | |  |  |  |  |  |  |  |  |  |  |
| 1 (least deprived) | | 1 |  |  | 1 |  |  | 1 |  | 1 |  |
| 2 | | **0.877 [0.826-0.931]** | **<.0001** |  | **0.931 [0.882-0.983]** | **0.0098** |  | **0.940 [0.894-0.988]** | **0.0015** | **0.918 [0.892-0.945]** | **<.0001** |
| 3 | | **0.912 [0.860-0.968]** | **0.0025** |  | 0.976 [0.926-1.030] | 0.3796 |  | **0.946 [0.900-0.994]** | **0.0268** | **0.910 [0.884-0.937]** | **<.0001** |
| 4 | | **0.896 [0.844-0.951]** | **0.0003** |  | 0.967 [0.917-1.019] | 0.2122 |  | **0.932 [0.887-0.979]** | **0.0005** | **0.899 [0.873-0.925]** | **<.0001** |
| 5 (most deprived) | | **0.885 [0.833-0.941]** | **<.0001** |  | **0.934 [0.885-0.986]** | **0.0137** |  | **0.923 [0.878-0.970]** | **0.0015** | **0.878 [0.853-0.904]** | **<.0001** |
| *Psychiatric history* | |  |  |  |  |  |  |  |  |  |  |
| No | | 1 |  |  | 1 |  |  | 1 |  | 1 |  |
| Yes | | **19.287 [18.628-19.970]** | **<.0001** |  | **9.883 [9.585-10.191]** | **<.0001** |  | **5.050 [4.899-5.205]** | **<.0001** | **1.762 [1.726-1.800]** | **<.0001** |
| *Duration of reference hospitalization (days) - mean(SD)* | | 0.998 [0.995-1.002] | 0.3020 |  | **1.010 [1.009-1.012]** | **<.0001** |  | **1.021 [1.020-1.022]** | **<.0001** | **1.018 [1.018-1.019]** | **<.0001** |
| *Level of clinical care* | |  |  |  |  |  |  |  |  |  |  |
| General hospital ward (medical, surgery, obstetrics) | | 1 |  |  | 1 |  |  | 1 |  | 1 |  |
| Intensive care unit | | **1.876 [1.757-2.002]** | **<.0001** |  | **1.134 [1.079-1.191]** | **<.0001** |  | 0.961 [0.921-1.002] | 0.0606 | **0.755 [0.734-0.776]** | **<.0001** |
| Intensive care unit with  invasive procedures | | **3.406 [3.064-3.785]** | **<.0001** |  | **2.124 [1.977-2.281]** | **<.0001** |  | **1.113 [1.040-1.190]** | **0.0019** | **0.511 [0.472-0.553]** | **<.0001** |
| * *Odds-ratio* adjusted for socio-demographic characteristics (sex, region and social deprivation index), psychiatric history and characteristics of the reference hospitalization : duration of hospitalization (days) and level of clinical care | | | | | | | | | | | |
